# Antiviral activity of luteolin against porcine epidemic diarrhea virus in silico and in vitro

**DOI:** 10.1186/s12917-024-04053-4

**Published:** 2024-07-03

**Authors:** Jieru Wang, Xiaoyu Zeng, Jiaojiao Gou, Xiaojie Zhu, Dongdong Yin, Lei Yin, Xuehuai Shen, Yin Dai, Xiaocheng Pan

**Affiliations:** 1 Anhui Province Key Laboratory of Livestock and Poultry Product Safety Engineering, Livestock and Poultry Epidemic Diseases Research Center of Anhui Province, Key Laboratory of Pig Molecular Quantitative Genetics of Anhui Academy of Agricultural Sciences, Institute of Animal Husbandry and Veterinary Sciences, Anhui Academy of Agricultural Sciences, Hefei, Anhui 230031 China; 2 China Institute of Veterinary Drug Control, Beijing, 100000 China

**Keywords:** PEDV, Luteolin, Porcine ACE2, Spike, Mpro, Pro-inflammatory cytokine, Drug resistant mutant

## Abstract

**Background:**

Porcine epidemic diarrhea virus (PEDV) mainly causes acute and severe porcine epidemic diarrhea (PED), and is highly fatal in neonatal piglets. No reliable therapeutics against the infection exist, which poses a major global health issue for piglets. Luteolin is a flavonoid with anti-viral activity toward several viruses.

**Results:**

We evaluated anti-viral effects of luteolin in PEDV-infected Vero and IPEC-J2 cells, and identified IC_50_ values of 23.87 µM and 68.5 µM, respectively. And found PEDV internalization, replication and release were significantly reduced upon luteolin treatment. As luteolin could bind to human ACE2 and SARS-CoV-2 main protease (Mpro) to contribute viral entry, we first identified that luteolin shares the same core binding site on pACE2 with PEDV-S by molecular docking and exhibited positive pACE2 binding with an affinity constant of 71.6 µM at dose-dependent increases by surface plasmon resonance (SPR) assay. However, pACE2 was incapable of binding to PEDV-S1. Therefore, luteolin inhibited PEDV internalization independent of PEDV-S binding to pACE2. Moreover, luteolin was firmly embedded in the groove of active pocket of Mpro in a three-dimensional docking model, and fluorescence resonance energy transfer (FRET) assays confirmed that luteolin inhibited PEDV Mpro activity. In addition, we also observed PEDV-induced pro-inflammatory cytokine inhibition and Nrf2-induced HO-1 expression. Finally, a drug resistant mutant was isolated after 10 cell culture passages concomitant with increasing luteolin concentrations, with reduced PEDV susceptibility to luteolin identified at passage 10.

**Conclusions:**

Our results push forward that anti-PEDV mechanisms and resistant-PEDV properties for luteolin, which may be used to combat PED.

**Supplementary Information:**

The online version contains supplementary material available at 10.1186/s12917-024-04053-4.

## Background

Porcine epidemic diarrhea (PED) is caused by porcine epidemic diarrhea virus (PEDV) and is one of the most devastating diseases in the global pig industry due to high mortality rates in piglets [1]. PEDV mutations and the incomplete protection afforded by vaccines (even against the unmutated virus) led to vaccine hesitancy that allowed the virus to adapt and continue to spread and cause illness [2]. Therefore, there is an increasing interest in developing effective anti-viral therapies to control PEDV infections.

PEDV belongs to alphacoronavirus (α-CoV) which has a positive single-stranded RNA genome encoding four structural proteins (spike, envelope, membrane, and nucleocapsid), accessory proteins, and two non-structural proteins (pp1a and pp1ab), which are processed into 16 non-structural proteins via multiple viral proteinases [3]. Main protease (Mpro) is one such viral protease which digests precursor polyproteins at 11 sites [4]. It was reported that Mpro was a high-profile target for anti-viral drug discovery in severe acute respiratory syndrome coronavirus 2 (SARS-CoV-2) [5, 6]. Besides, spike protein (S) mediated PEDV entry by binding of the host cell receptor. Blocking S protein and receptor recognition is also a potential solution for anti-viral drug development [7]. Therefore, anti-viral drugs targeting Mpro or S protein may be promising pathways to fight PEDV infections.

New anti-viral compounds from natural origins can be used to develop effective therapeutic agents against PEDV infections. Flavonoids were reported as phytotherapeutics to combat cytokine storms in SARS-CoV-2 [8]. Luteolin is a common bioflavonoid found in a variety of fruits and vegetables [9], and possesses several beneficial medicinal properties, such as anti-tumour, anti-inflammatory, cardio-protective, and neuroprotective effects [10]. Luteolin also reportedly exerts inhibitory effects toward Dengue, Epstein-Barr (EB), Japanese encephalitis, hepatitis B, and influenza A viruses [11–14]. Inhibitory mechanisms include the activation of an extracellular signal-regulated kinase, the down-regulation of hepatocyte nuclear factor 4, inhibition of the host pro-protein convertase furin, and repression of EB-induced immediate-early genes [15–17]. Luteolin also specifically binds to the S-angiotensin-converting enzyme 2 (ACE2) protein interactions in SARS-CoV-2 to block viral entry into host cells and inhibit SARS-CoV Mpro enzymatic activity [7, 18, 19]. Furthermore, luteolin and the luteolin structural analog eriodictyol were previously identified as potential treatments for SARS-CoV-2[8]. Luteolin also inhibits cytokine storms caused by interleukin (IL)-1b and histamine production csex:/bookmark in mast cells upon SARS-CoV-2 stimulation [7]. Due to these properties, interest in potential luteolin mechanisms in PEDV infection has increased. In this study, we investigated luteolin effects on the PEDV replication cycle, anti-PEDV mechanisms and luteolin-resistant PEDV properties. We provide novel insights into the potential use of luteolin as an anti-PEDV therapeutic, which may benefit the swine industry.

## Results

### The evaluation of luteolin cytotoxicity on Vero and IPEC-J2 cell lines

**Fig. 1:**
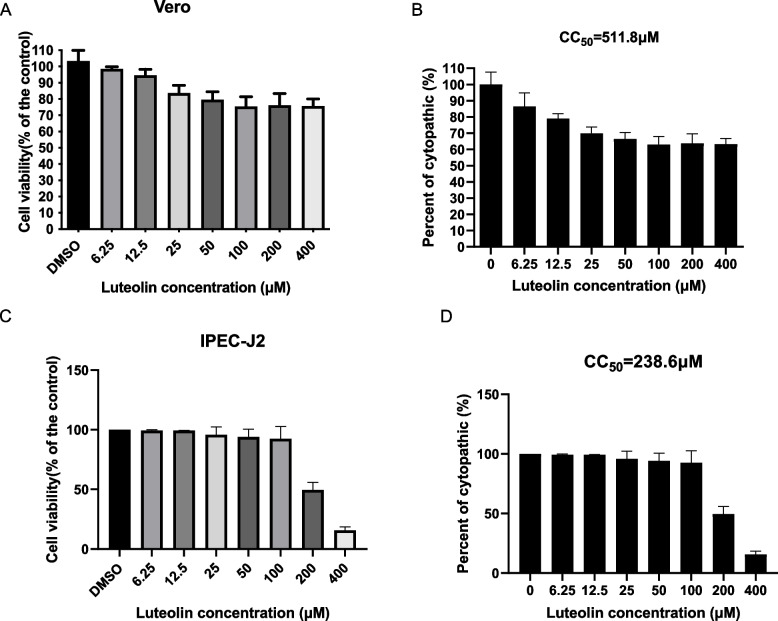
The evaluation of luteolin cytotoxicity on Vero and IPEC-J2 cell lines. **A-B** The luteolin cytotoxicity on Vero (**A**) and IPEC-J2 (**B**) cell lines by CCK-8 assays. **C-D** The CC50 values were calculated based on CCK-8 data of Vero (**C**) and IPEC-J2 (**D**) cell lines by nonlinear regression analysis with GraphPad Prism v.9.0.0

We first measured luteolin-mediated cytotoxicity in Vero cells by CCK8 assay, the cell viability was more than 80% when the concentration of luteolin was greater than 25 µM (Fig. 1A). and the 50% cytotoxic concentration (CC_50_ value) was calculated at 511.8 µM with nonlinear regression analysis (Fig. 1B). These luteolin cytotoxicity were further verified in IPEC-J2 cells by determining CC_50._ Consistent with luteolin cytotoxicity in Vero cells, the CC_50_ value of luteolin was 238.6 µM (Fig. 1C and D).

### Anti-viral activity of Luteolin on PEDV infected Vero, and IPEC-J2 cells

**Fig. 2:**
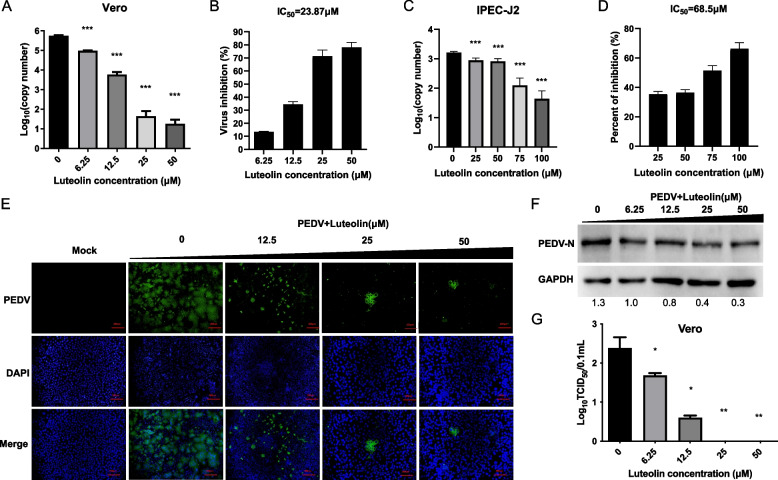
Anti-viral activity of Luteolin on PEDV infected Vero, and IPEC-J2 cells. **A** The anti-PEDV effects of luteolin in Vero cells by using absolute RT-PCR assay. **B** IC_50_ values in Vero cells were calculated based on RT-PCR data. **C** Anti-PEDV effects of luteolin by using RT-PCR assay in IPEC-J2 cells. **D** IC_50_ values in IPEC-J2 cells were calculated based on RT-PCR data. **E–G** The anti-PEDV effects of luteolin in Vero cells by using immunofluorescence assay (**E**), western blot (**F**) and TCID_50_ assays (**G**). Vero cells infected with PEDV and cultured with different concentrations of luteolin (0-50 μM). Densitometry quantification immunoblot analysis results of PEDV-N presented relative to those of GAPDH ﻿using Image J software. Values represent the mean ± standard deviation from three independent experiments. ns: non-significant; *P < 0.05; **P < 0.01; ***P < 0.001

To investigate the anti-viral effects of luteolin, cells were treated with twofold serial dilutions of luteolin after cells were infected with PEDV for 1 h. One days later, cytopathic effects (CPEs) were obvious in DMSO-treated Vero cells, but cells treated with high-dose luteolin remained morphologically unchanged. Viral copy number due to luteolin dilutions were observed and PEDV copy number gradually reduced with increasing luteolin concentrations in Fig. 2A. The IC_50_ value for luteolin was 23.87 µM (Fig. 2B). The selectivity index (CC_50_/IC_50_) for luteolin was 21.44, which indicated that luteolin had potential anti-PEDV activities. In addition, the anti-viral effects were further verified in IPEC-J2 cells by determining IC_50_ values. Consistent with anti-viral activity in Vero cells, the IC_50_ value was 68.5 µM (Fig. 2C and D), and the selectivity index (CC_50_/IC_50_) value was 3.48. Thus, luteolin had conserved roles across species.

To further investigate the anti-viral effects of luteolin in PEDV-infected Vero cells, we performed the indirect immunofluorescent assay (IFA) and western blot (WB) assays on the PEDV-N protein. As shown in Fig. 2E and F, with increasing luteolin concentrations, N protein gradually reduced in cells, indicating a luteolin-mediated dose-dependent inhibition of PEDV replication. Luteolin at 25 µM significantly inhibited PEDV replication when compared with control cells. Titration results were similar. As shown in Fig. 2G, as the luteolin concentration reached 25 µM, virus yields were not detected in infected cells. Thus, luteolin appeared to mediate a dose-dependent inhibition of PEDV replication in Vero cells.

### The effect of luteolin in different viral replication steps

**Fig. 3:**
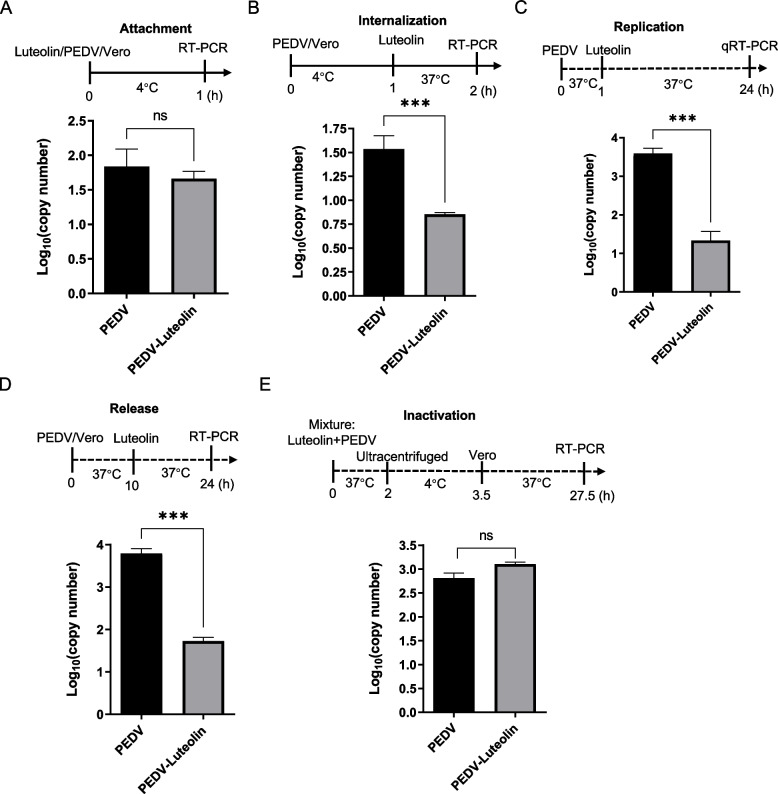
The effect of luteolin in different viral replication steps. The effects of luteolin on PEDV attachment (**A**), internalization (**B**), replication (**C**), release (**D**), and inactivation (**E**) in Vero cells (RT-PCR assay data). Values represent the mean ± standard deviation from three independent experiments. ns: non-significant; *P < 0.05; **P < 0.01; ***P < 0.001

To ascertain which virus life-cycle step luteolin was most effective at, we supplemented luteolin to cells at different times in the PEDV infection cycle. In viral attachment assays, no significant differences in PEDV RNA levels were observed between luteolin-treated and DMSO-treated cells (Fig. 3A). However, luteolin administration (25 µM) at internalization, replication, and release inhibited the virus, as reflected by significantly lower copy numbers when compared with control group (Fig. 3B–D). Additionally, luteolin interacted with PEDV particles, although virus pre-exposure to luteolin unaltered PEDV particle infectivity (Fig. 3E). Therefore, luteolin administration at virus internalization, replication, and release steps generated was highly effective.

### Luteolin inhibits PEDV internalization independent of PEDV-S binding to pACE2

**Fig. 4:**
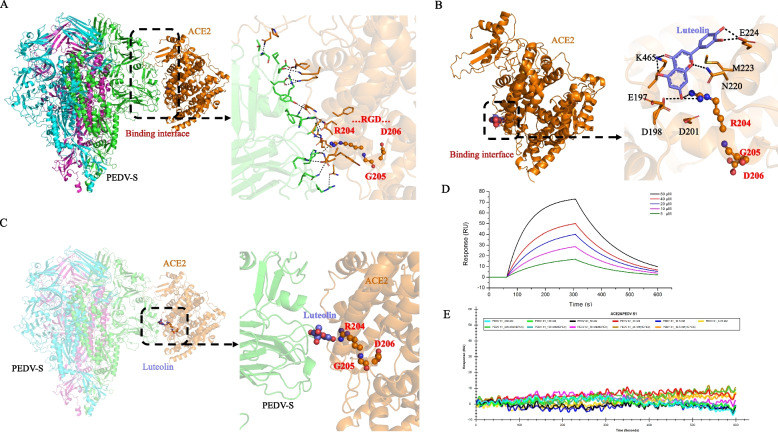
Luteolin inhibits PEDV replication independent of PEDV-S binding to pACE2. **A** Molecular docking data show interactions between PEDV-S and pACE2. **B** Molecular docking data show luteolin interactions with pACE2. **C** Luteolin binds to the same position on the PEDV-S surface as the PEDV-S/pACE2 binding interface. **D** Luteolin binding curve with pACE2; 5 µM–80 µM luteolin and 200 nM pACE2 were used. **E** Binding curve of pACE2 with PEDV-S1. pACE2, captured on a COOH chip, did not bind different PEDV-S1 concentrations (12.5–200 nM)

Luteolin had been shown to inhibit SARS-CoV-2 S–ACE2 interactions by directly binding to human ACE2 [7]. As CoV family member, we hypothesized that PEDV-S was blocked outside the cells by luteolin in the same way. Therefore, amino acid residues at the interaction interface were observed using a three-dimensional docking model of the PEDV-S/pACE2 complex (Fig. 4A). According to hydrogen bond binding modes and the complementarity of hydrophobic/hydrophilic properties on PEDV-S and pACE2 molecular surfaces (Fig. 4A right sections), one hot spot, including residues R204-G205-D206, was identified as the core region which stabilized the complex. Furthermore, the core binding region also had good binding affinity (− 7.92 kcal/mol) with luteolin as, and one residue R204 was identified as forming a direct hydrogen bond with the oxhydryl group on luteolin (Fig. 4B). Thus, the luteolin ligand shared the same core binding site on pACE2 with PEDV-S (Fig. 4C). Luteolin also showed positive pACE2 binding, with an affinity constant of 71.6 µM in dose-dependent increases (Fig. 4D) by SPR assay. However, pACE2 was incapable of binding to 12.5-200 nM PEDV-S1 (Fig. 4E). Therefore, luteolin inhibited PEDV internalization independent of PEDV-S binding to pACE2.

### Luteolin inhibits PEDV Mpro enzymatic activity to diminished PEDV replication

**Fig. 5:**
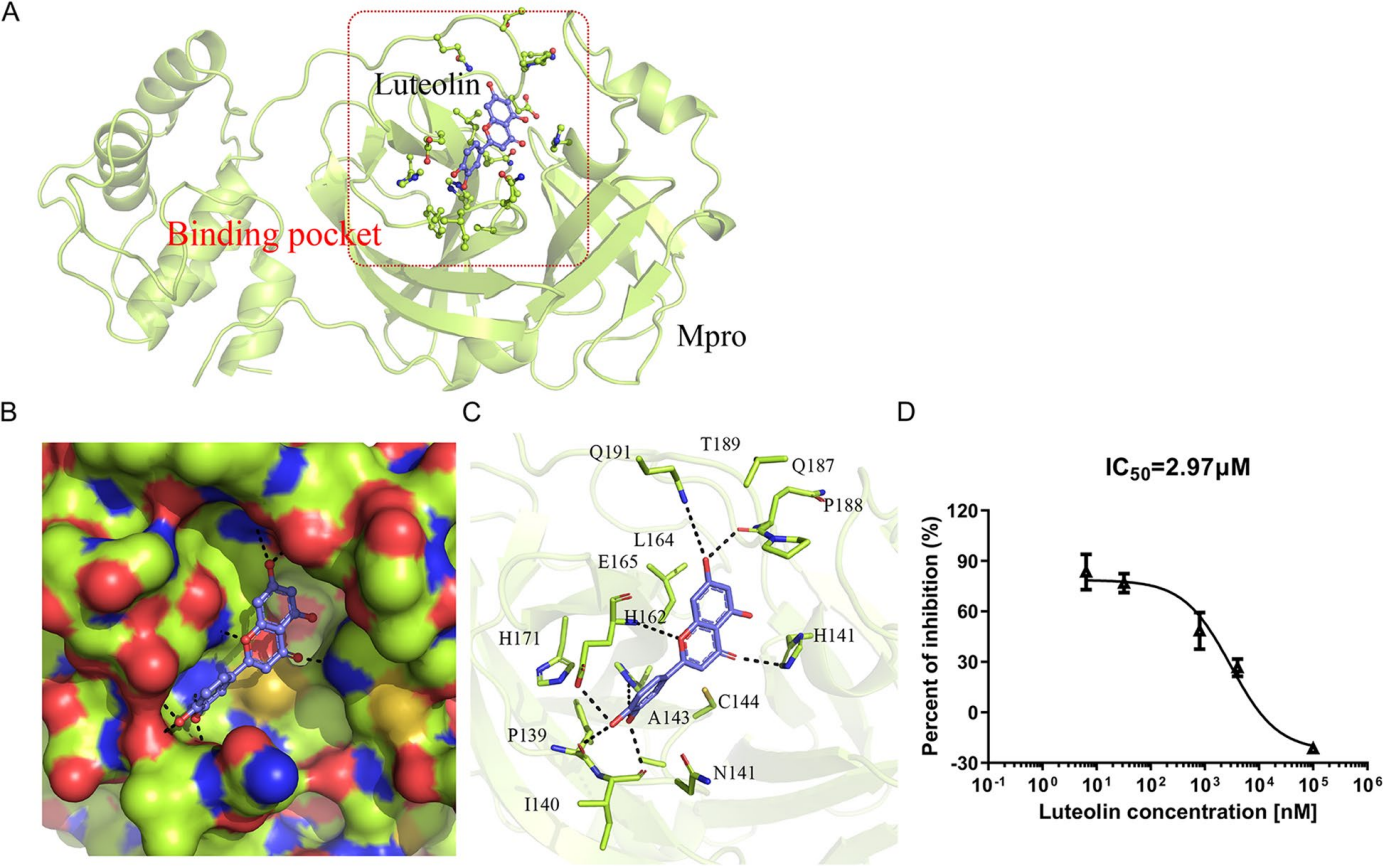
Binding mode between Mpro and luteolin. **A** Binding patterns between Mpro (PDB: 7W6M) and luteolin. Red box: the candidate binding pocket in Mpro; **B** Hydrophilic-hydrophobic interactions between luteolin and Mpro in the binding pocket; **C** Key Mpro residues form binding interactions with the luteolin ligand. The binding free energy between Mpro and luteolin was − 7.36 kcal/mol. **D** Luteolin effects on Mpro activity. The IC_50_ was determined in GraphPad. Data are shown as the mean ± standard deviation from three independent experiments

We previously observed that the flavonoid wogonin bound to the PEDV Mpro protein by inhibiting its enzymatic activity in order to regulate PEDV replication [20]. Here, for luteolin, molecular docking was performed to determine potential binding sites and binding affinity between Mpro (PDB: 7W6M) and luteolin. Docking results indicated that luteolin fit into a common binding pocket in Mpro (Fig. 5A and B). Luteolin displayed good binding affinity to Mpro, with an estimated free binding energy of − 7.36 kcal/mol. Polar interactions played the major role in stabilizing binding ability for protein–ligand complex. The ligand formed strong hydrogen bonds with surrounding polar amino acids in the pocket, including H141, H162, E165, and Q191. Also, the dihydroxyl group on the luteolin ligand formed hydrogen bonds with − NH and − C = O groups on the backbones of P139, I140, and Q187 residues. Additionally, hydrophobic interactions were important in complex stabilization. they were observed between benzene rings on luteolin and on adjacent A143, C144, and L164 residues (Fig. 5C).

Subsequently, we assessed the effect of luteolin on enzymatic activity of recombinant Mpro protein by using FRET assay, which had been validated in our previous reports [21]. Luteolin addition led to a concentration-dependent inhibition of purified PEDV Mpro activity, with an IC_50_ = 2.97 µM (Fig. 5D). Thus, luteolin exerted inhibitory effects toward PEDV Mpro.

### Luteolin inhibits PEDV-induced inflammatory response

**Fig. 6:**
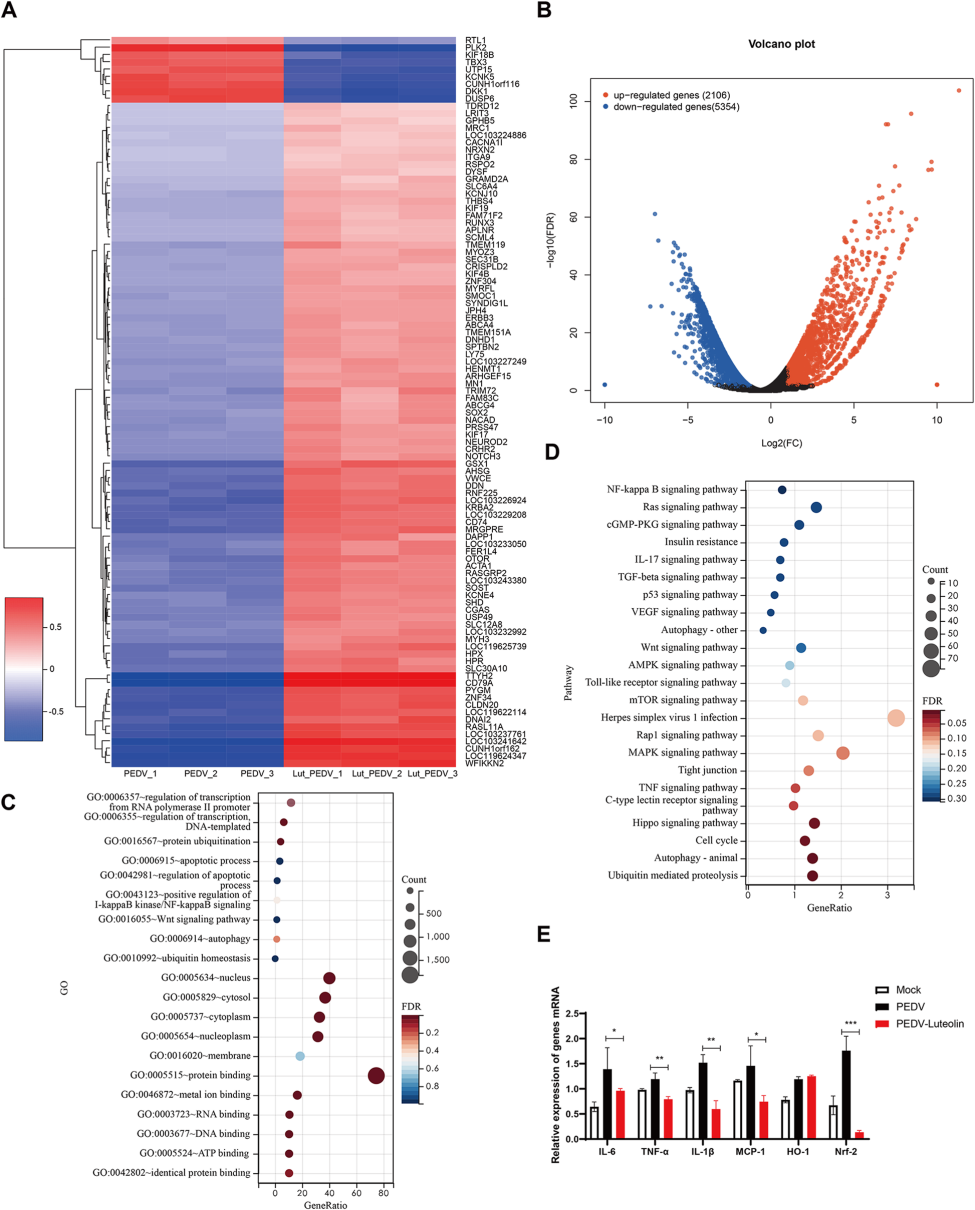
Luteolin effects on pro-inflammatory cytokine levels. Overview of differentially expressed genes (DEGs) in PEDV and luteolin + PEDV groups. **A** The heat map shows DEGs convergence in different groups. **B** Volcano Plot showing DEPs. Cut off ratio of 1.5-fold and a *P*-value ≤ 0.05. **C-D** DEGs analysis between luteolin + PEDV and PEDV groups using GO (**C**) and KEGG (**D**) databases. **E** RT-PCR analysis of IL-6, IL-1β, TNF-a, MCP1, HO-1, and Nrf2 expression levels in different groups. Values represent the mean ± standard deviation from three independent experiments. ns: non-significant; *P < 0.05; **P < 0.01; ***P < 0.001

To explore luteolin mechanisms during PEDV infection, transcriptomics was performed on Vero cells in PEDV and luteolin + PEDV groups. Heat maps of quantified genes displayed distinct and specific expression patterns (Fig. 6A). The luteolin + PEDV group contained 2,106 up-regulated and 5,354 down-regulated genes compared with the PEDV group (Fig. 6B). Then, DEGs were annotated using the Gene Ontology database. Genes mostly affected by PEDV infection were mainly involved in transcription, protein ubiquitination, and apoptotic processes (Fig. 6C). Pathway analyses showed that most DEGs were related to nuclear factor κB (NF-κB) and mitogen-activated protein kinase (MAPK) signaling pathways (Fig. 6D). Therefore, we compared inflammatory factor levels in the absence/ presence of luteolin during PEDV infection. When compared with the PEDV group, luteolin administration significantly reduced IL-6, IL-1β, TNF-a, and MCP1/2 levels, which were increased by PEDV infection (Fig. 6E). Heme oxygenase 1 (HO-1) is an inducible enzyme with anti-inflammatory effects, and has critical roles in host defenses against viral infections [22]. As shown in Fig. 6E, Luteolin treatment was unable to reduce the PEDV induced HO-1 expression in Vero cells. Nrf2 has important roles in HO-1 induction in many cell types [21]. An addition of luteolin markedly lowered (*p* < 0.05) Nrf2 expression. These results demonstrate that luteolin suppresses the production of pro-inflammatory cytokines induced by PEDV infection.

### The ability of PEDV to generate resistance against luteolin

**Fig. 7:**
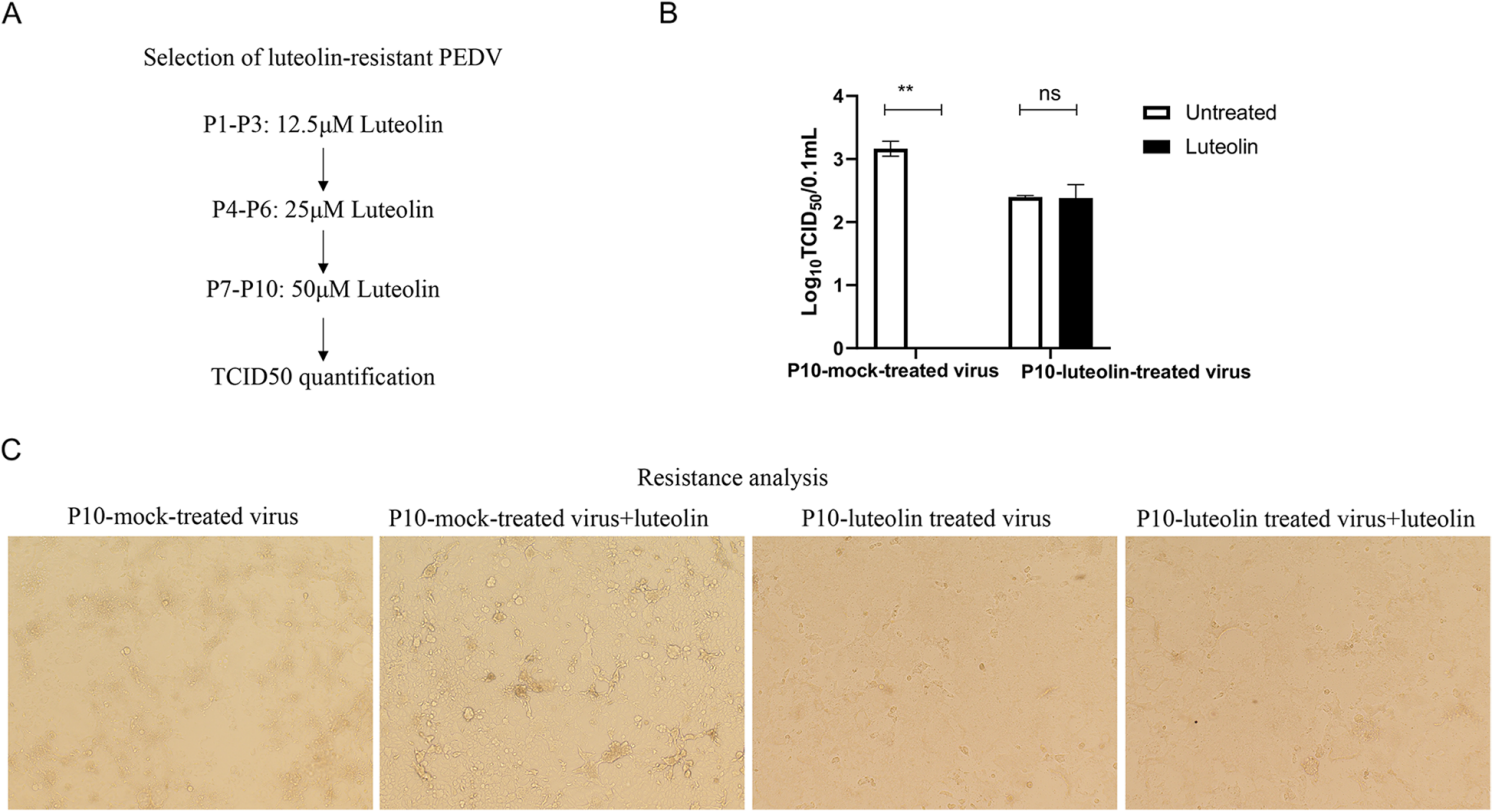
Luteolin-resistant PEDV is generated after 10 passages. **A** Scheme showing the selection of a luteolin-resistant PEDV mutant virus. P1–P3 passages were subjected to 12.5 μM luteolin, P4–P6 to 25 μM luteolin, and P7–P10 to 50 μM luteolin. **B** Resistance analysis. Vero cells were infected with P10 mock-treated or luteolin treated virus at MOI = 0.05 for 1 h followed by 48 h incubation in media plus 50 μM luteolin or DMSO (negative control). Viral titers were quantified by TCID_50_ assays and resistance quantified by comparing viral titers between luteolin-treated and luteolin untreated infections. Values represent the mean ± standard deviation from three independent experiments. ns: non-significant; *P < 0.05; **P < 0.01; ***P < 0.001

Since Mpro is the virus protein, we examined if it is an attractive therapeutic target that will not readily lead to escape mutants. We generated resistant viruses by passaging the PEDV AH2012/12 strain in Vero cells in the presence of increasing luteolin concentrations (Fig. 7A). Viruses from passage 10 (P10) were assayed for a resistance phenotype by comparing viral titers between mock- and luteolin-treated infections. When Vero cells were infected at an MOI = 0.05, the viral titer yield from the P10 virus was 1.3-fold lower than the wild-type, suggesting that a reduced viral fitness from this passage may have reflected the emergence of resistance mutations. Indeed, P10 virus infectivity in the presence/absence of 50 μM luteolin was approximately the same (Fig. 7B and C), suggesting that this virus had acquired resistance to luteolin during cell culture.

## Discussion

Luteolin is a natural flavonoid extensively present in fruit and vegetables, such as chrysanthemum flowers, onion leaves, celery, parsley, carrots, sweet bell peppers, and broccoli [9]. Luteolin possesses not only anti-inflammatory activities and anticancer but also antiviral activities [8]. It appears to particularly target several porcine viruses, such as influenza [12], Japanese encephalitis virus [14], Pseudorabies Virus [17], Foot-and-mouth-disease virus [23] and African swine fever [24]. Here, we observed that luteolin exerted strong inhibitory effects on PEDV in vitro, with IC_50_ values of 23.87 µM and 68.5 µM in Vero and IPEC-J2 cells, respectively. The different anti-viral effects of luteolin on PEDV in the two cells may be due to the different infection mechanisms of PEDV in different cells. ﻿Vero cells do not express type I interferon [25]. Viral replication may be intricately regulated by multiple signaling pathways. In another study, Choi et al. observed that luteolin exhibited an IC_50_ activity of < 12 µg/mL, consistent with our results, but did not explore its anti-viral mechanisms [26]. In other work, a phytosomal luteolin formulation (in olive pomace oil-NeuroProtek®) effectively improved autism spectrum disorder-the liposomal formulation not only improved oral absorption and bioavailability, but also provided additional neuroprotective and anti-inflammatory actions [27]. Therefore, luteolin has broad-spectrum antiviral potential in the pig industry.

To comprehensively elucidate luteolin actions on PEDV multiplication, we investigated luteolin effects on PEDV infection cycle steps. Luteolin did not interact with PEDV particles, as virus pre-exposure to luteolin did not alter PEDV particle infectivity. However, luteolin inhibited PEDV internalization, replication, and release, but not attachment, suggesting that the mode of action occurred after PEDV attachment. It was reported that luteolin interrupted the interaction of cell surface furin protein and SARS-CoV-2 S protein, which rely on proteolytic cleavage by host proteases to facilitate fusion priming/activation [18]. However, the PEDV-S2 protein lacks the cutting site of furin[16]. Hence, we focused on the ACE2 protein, which was reported that luteolin blocked SARS-CoV-2 cell attachment as a competitive inhibitor of ACE2-S protein [7]. Even though we identified the ligand luteolin shares the same core binding site on pACE2 with PEDV-S based on the three-dimensional docking model of the PEDV-S/pACE2 complex, and observed the luteolin showed positive pACE2 binding with the affinity constant of 71.6 µM in dose-dependent increase. However, we identified that pACE2 was incapable of binding to PEDV-S1 by SPR. Therefore, luteolin inhibited PEDV internalization independent of PEDV-S1 binding to pACE2. It was likely that pACE2 was not a receptor for PEDV invaders, although PEDV infection significantly upregulated the CoV receptor (ACE2) in enterocytes [28] and ACE2 bound to the CoV binding domain of integrin [29].

Moreover, in vitro enzyme inhibition studies showed that luteolin inhibited CoVs Mpro proteins [30–32]. Highly conserved CoVs Mpro is a promising and specific drug target because it is vital for cleavage, and has unique cleavage sites not present in human host proteases [32]. Additionally, luteolin may function as a potential phytotherapeutic to combat cytokine storms during SARS-CoV-2 infections [33, 34]. Therefore, we examined if luteolin inhibited PEDV replication by blocking the enzyme digestion activity site of Mpro, or counteracted the PEDV-mediated elevated levels of inflammatory cytokines levels. Here, we found that luteolin was firmly embedded in the groove of the active pocket of PEDV Mpro by using in vitro and in silico approaches. And luteolin significantly reduced PEDV-induced pro-inflammatory cytokines except for HO-1. Hence, luteolin inhibited PEDV replication by targeting Mpro and anti-inflammatory activities.

It is accepted that the virus is passaged mutated during drug use, leading to expanded drug-resistant strains during anti-viral therapy. Since in vitro enzyme inhibition studies showed that luteolin inhibited both the host ACE2 and viral Mpro proteins, resistant mutants were generated in cell culture to provide further insights into the mechanism of action of the drug which could influence the specific use of luteolin medicines in PED. We isolated a drug resistant mutant after 10 passages in the presence of increasing luteolin concentrations, and identified PEDV with reduced susceptibility to luteolin at this passage. Peng et al. reported that luteolin-escaped dengue virus mutants were mapped to the precursor membrane protein on the virion surface, and that NS2B proteins and key mutation residues in dengue virus, induced by luteolin, occurred at the host–pathogen interface [13]. Since the onset of epidemic diarrhea in piglets is acute, short-course anti-viral treatments could be attractive therapeutic approaches targeting PEDV.

## Conclusion

Overall, we evaluated the anti-viral effects of luteolin in PEDV-infected Vero and IPEC-J2 cells and found PEDV internalization, replication and release were significantly reduced upon luteolin treatment. Luteolin exhibited positive pACE2 binding by SPR assay. However, pACE2 was incapable of binding to PEDV-S1. Therefore, luteolin inhibited PEDV internalization independent of PEDV-S binding to pACE2. FRET assays confirmed that luteolin inhibited PEDV Mpro activity. In addition, we also observed PEDV-induced pro-inflammatory cytokine inhibition and Nrf2-induced HO-1 expression. Finally, luteolin resistant PEDV generated after 10 passages. Our results push forward that the anti-PEDV mechanism of luteolin and luteolin resistant PEDV property, which could influence the specific use of luteolin in PED.

## Methods

### Cell culture and virus

African green monkey kidney epithelial cells (Vero-E6) ﻿were purchased from the Cell Bank of the Chinese Academy of Sciences (Shanghai, China). IPEC-J2 cells were kindly donated by Professor Li Bin of the Jiangsu Academy of Agricultural Sciences. Vero and porcine small intestinal epithelial cells (IPEC-J2) were maintained in Dulbecco’s modified eagle medium (DMEM; Gibco, USA) plus 10% fetal bovine serum (Natocor, Argentina), 100 U/mL penicillin, and 100 mg/mL streptomycin. The PEDV G2 strain AH2012/12 (GenBank accession No. KU646831).

### Cytotoxicity assay

Cell viability was assessed using cell counting kit-8 (CCK8) (Meibio, Beijing, China) based on manufacturer’s instructions. Each Vero cells and IPEC-J2 were incubated with different concentration of Luteolin (6.25–400 µM) in 96- well plate for 48 h. Plates were washed three times with phosphate buffered saline (PBS), The supernatant was replaced by 110 µL of DMEM that contain 9.09% CCK-8 reagent. Plates were incubated in the dark for 2 h. After this, cells were gently shaken and the optical density (OD450) were measured using a microplate reader (﻿Victor NIVO 3S, USA). Relative cell viability was calculated the luteolin fifty percent cytotoxic concentration (CC_50_) of was calculated with nonlinear regression analysis by GraphPad Prism v.9.0.0 based on previous research [20].

### Viral infection

To evaluate the anti-viral effects of luteolin in PEDV-infected Vero and IPEC-J2 cells. Vero cells were added to 12-well plates and inoculated with PEDV at a multiplicity of infection (MOI) = 0.01, along with 8 µg/mL trypsin in the absence/presence of luteolin (6.25–50 µM) for 1 h. For IPEC-J2 cells, cell infection processes were similar; the MOI = 0.1, but 5 µg/mL trypsin was used in the absence/presence of luteolin (25–100 µM). The different concentrations of trypsin added to Vero and IPEC-J2 cells was determined by the highest cell tolerance to trypsin and the lowest concentration of trypsin-assisted PEDV infection. After viral adsorption, cells were washed twice in PBS to get rid of the unabsorbed PEDV particle and cultured for 24 h. Cells were finally harvested for PEDV RNA detection by real-time reverse transcription PCR (RT-PCR), immunofluorescence assay (IFA), and median tissue culture infectious dose (TCID_50_) assay.

### Absolute quantitative RT-PCR

For measuring the half-maximal inhibitory concentration (IC_50_) of luteolin for PEDV infected cell lines, the total cellular RNA was isolated by TRIzol (Invitrogen) To demonstrate luteolin inhibitory effects on PEDV, cells were treated as described and collected the supernatant. 200 μL of supernatant for viral RNA extraction (Takara, #9766) following manufacturer’s instructions. cDNA was generated by reverse transcription using the Novo Script® Plus All-in-one 1st Strand cDNA Synthesis SuperMix9 kit (Cat No. E047). PEDV loads were measured using an AceQ® qPCR SYBR Green Master Mix kit (Cat No. Q111) using the following primers; PEDV-186-F (5′-TACTAAGCGTAACATCCTGCC-3′) and PEDV-186-R (5′-GTAGTACCAATAACAACCGAAGC-3′) for absolute RT-PCR with 40 cycles. The 186-base pair (bp) PEDV fragment of the *ORF1ab* gene (14,166 bp to 14,351 bp) was cloned into the pMD19-T vector and served as an internal reference for PEDV copy-number quantification.

The half-maximal inhibitory concentration (IC_50_) of luteolin was calculated. IC_50_ = (PEDV infected group-luteolin treatment and PEDV co-infected group)/(PEDV infected group -blank control group) × 100%, and finally the IC_50_ was calculated with nonlinear regression analysis by GraphPad Prism v.9.0.0. The therapeutic index was defined as CC_50_/IC_50_.

### Indirect immunofluorescent assay (IFA)

To evaluate the anti-viral effects of luteolin in PEDV-infected Vero cells. Cells were fixed in 4% paraformaldehyde for 20 min at room temperature. After washing three times in PBS, ice-cold 0.3% TritonX-100 was added and cells incubated for 10 min. 1% bovine serum albumin in PBS was used to block cells for 40–60 min at room temperature.Subsequently, an anti-PEDV-N protein antibody [35] was added for 2 h at room temperature. Finally, cells were incubated with Alexa Fluor®488 Donkey anti-Mouse IgG (Antgene, Wuhan, China) at room temperature for 1 h, washed with PBS, and treated with 4′,6-diamidino-2-phenylindole dihydrochloride (DAPI, Beyotime Biotechnology, Shanghai, China) for 15 min to stain nuclei.

### Western blot analysis

The cells were harvested in 100µL of lysis buffer, the lysate separated in 10% SDS-PAGE and transferred to a polyvinylidene difluoride (PVDF, Yeasen, #36124ES10) membrane. The membrane was blocked using 5% skimmed milk for 1 h and incubated overnight at 4 °C in the presence of specific primary antibodies, including anti-PEDV-N protein antibody and GAPDH pAb (Diaan, #3058). The membrane was reincubated with their corresponding secondary HRP-labelled antibodies at a dilution of 1:4000 for 2 h at room temperature. The membranes were later washed using a TBST buffer and analysed by film exposure after enhanced chemiluminescence (ECL) reaction.

### TCID_50_ assay for measuring PEDV titers

The harvested virus suspension was serially tenfold diluted and used to inoculate confluent Vero cell monolayers in 96-well plates. The infectious dose 50% (TCID_50_) in units/mL of supernatant was calculated according to the Reed–Muench method [36].

### The effect of luteolin on PEDV life cycle

To determine luteolin effects on PEDV attachment, Vero cells were infected with PEDV (MOI = 0.05) in the absence/presence of luteolin (25 μM) at 4 °C for 1 h. After washing the unabsorbed virus three times in PBS, cell lysates were harvested for absolute quantitative RT-PCR.

For internalization assays, Vero cells were infected with PEDV (MOI = 0.05) for 1 h at 4 °C. Unbound viruses were removed by washing three times in PBS, and cells were incubated at 37 °C for 2 h in media plus 25 μM luteolin. Cell lysates were then harvested for absolute quantitative RT-PCR.

For replication assays, Vero cells were infected with PEDV (MOI = 0.05) for 1 h at 37 °C. Unbound viruses were removed by washing three times in PBS, and cells were incubated with luteolin (25 μM) at 37 °C for 24 h. Cell lysates were harvested for absolute quantitative RT-PCR.

For PEDV release assays, Vero cells were infected with PEDV (MOI = 0.05) for 10 h at 37 °C. Cells were washed three times in PBS and incubated with luteolin (25 μM) at 37 °C for 14 h. Supernatants were harvested for absolute quantitative RT-PCR.

For PEDV inactivation assays, PEDV (MOI = 0.05) and luteolin (25 μM) were incubated at 37 °C for 2 h. The mixture supernatant was topped up with PBS and ultracentrifuged at 90,000 × g for 1.5 h at 4 °C in 20% sucrose buffer (w/w) to purify virus and then was used to infect Vero cells for 24 h. Cell lysates were harvested for absolute quantitative RT-PCR [37].

### Homology modeling and structure refinement of Mpro, porcine ACE2 (pACE2) and PEDV-S

To evaluate the interaction between luteolin with PEDV Mpro, Spike proteins (PEDV-S) and pACE2. The crystal structures of PEDV Mpro and S protein were previously resolved (RCSB PDB ID: 4XFQ, https://www.rcsb.org/structure/4XFQ and 7W6M, https://www.rcsb.org/structure/7W6M) onto the RSCB Brookhaven Protein Databank (PDB) database, while the pACE2 protein (*Sus scrofa*) was constructed using the homology modeling method. The pACE2 amino acid sequence was extracted from the National Center for Biotechnology Information database (NP_001116542.1) and used to search against PDB, from which the minke whale pACE2 protein (RCSB PDB ID: 7WSF), with 90.66% sequence identity, was identified as a template structure for homology modeling. The modeled three-dimensional structure with the lowest discrete optimized protein energy score was chosen, and a > 5 ns molecular dynamic simulation energy was minimized in Amber16 software using an AMBER FF14SB force field.

### Molecular docking of pACE2 and PEDV-S

For predicting the optimal binding configuration between luteolin with pACE2 and PEDV-S, a rigid protein–protein docking simulation was used to evaluate binding modes between pACE2 and PEDV-S by using Rosetta Dock software. The docking process started with the score_jd2 program which added hydrogen atoms. Then, 20 docking runs were performed, each yielding up to 2,500 models for a maximum of 50,000 models/constraint combination. For comparison purposes, 20 docking runs, each yielding 5,000 models (100,000 in total), were performed without constraints. For each constraint combination and for unconstrained docking exercises, the top-scoring docking complex for pACE2/PEDV-S was identified as a representative model for analysis.

### Flexible docking of the proteins and luteolin

To clarify interaction of luteolin and other protein with 3D Structure, the 3S chemical structure of luteolin was drawn in ChemDraw and optimized using Chem3D 21 software. Docking simulations with luteolin were performed in AutoDock4.0 with Mpro, ACE2, and PEDV-S proteins using a Lamarkian genetic algorithm. In total, 50 docking conformations were obtained and ranked according to docking energy values; the top scoring conformation with the lowest binding energy was selected for binding mode analysis.

### Fluorescence Resonance Energy Transfer (FRET) assay for enzymatic characteristics

To evaluate whether luteolin affects the enzymatic characteristics of Mpro, the FRET assay was performed with a purified Mpro protein with ability of effectively cleave the fluorogenic peptide substrate based on our previous report [20]. Then, 8 µg/mL Mpro was pre-incubated with 1.28 nM–100 µM luteolin at 37 °C for 1 h, after which, 10 µM of Dabcyl-YNSTLQ↓AGLRKM-E-Edans substrate peptides were added at 37 °C for 1 h to test luteolin inhibitory effects toward PEDV Mpro. A reaction system without luteolin was used as a control. Fluorescence emitted by the cleaved substrate was measured using a SpectraMax M5 Microplate Reader (340 nm excitation and 485 nm emission). The inhibition ratio was calculated using the following equation: percentage inhibition (%) = 100 × [1-fluorescence of the experimental group/the control group].

### Surface plasmon resonance assay

To evaluate the interaction between pACE2 and PEDV-S protein, the recombinant pACE2 his-tagged protein (R&D, #10545) was immobilized on activated COOH-sensor chips, which served as a ligand, on an OpenSPR system (Nicoya Lifesciences Inc., Kitchener, ON, Canada). Then, 5–80 μM of luteolin or recombinant PEDV-S1 protein (Novoprotein, #DRA1646, 26–200 nM) was added to the sensor chip using PBS as a running buffer. Kinetic constants, including the association constant (ka), kd, and the affinity constant (KD = kd/ka), were calculated according to a 1:1 binding model.

### RNA sequencing and transcriptome analysis

To explore luteolin mechanisms during PEDV infection, RNA libraries were created from luteolin treated Vero cell infected with PEDV and PEDV Infected Vero cell only groups by high-throughput RNA-seq. Three replicates were conducted per sample. Total RNA was isolated from samples and mRNA were enriched by oligo (dT) tagged magnetic bead. Library construction and sequencing were performed using MGISEQ-2000RS. Sample sequence quality was assessed using FastQC (v.0.11.7), and quality trimming was conducted using the FASTX-Toolkit (v.0.0.14) to remove bases with Phred33 scores < 30, while retaining reads of at least 50 base pairs in length. Quality trimmed reads were mapped against the *S. scrofa* reference genome using HISAT2 (v.2.1.0). Gene expression profiling was performed based on the number of reads. Fragments per kilo base of exon model per million mapped read values from each uni gene were used to estimate expression values and transcript levels in SAMtools (v.1.7) and HTSeqcount (v.0.9.1).

### Relative quantitative RT-PCR

To assess luteolin effects on PEDV-infected Vero cells, the mRNA expression of several inflammatory factors containing IL-6, tumor necrosis factor-α (TNF-α), IL-1β, monocyte chemotactic protein-1 (MCP-1), heme oxygenase-1 (HO-1) and nuclear factor erythroid 2-related factor 2 (Nrf-2), were tested before and after PEDV infection by relative quantitative RT-PCR. The total cellular RNA was isolated by TRIzol (Invitrogen). All primers are listed in Table 1.

**Table 1:** Pro-inflammatory cytokine primers used in this study

**Name**	**Forward primer (3**'**-5**'**)**	**Upstream primer (3**'**-5**'**)**
IL-6	AAAAGGTGGGTGTGTCCTCC	TGGCATTGCATCCCTGAGTT
TNF-α	TAGGCTGTTCCCACATAGCC	GGCGATTACAGACACAACTCC
IL-1β	TCCAGGGACAGGATCTGGAG	AACACGCAGGACAGGTACAG
MCP-1	CTCGCTCAGCCAGATGCAAT	CTTCTATAGCTCGCCAGCCTC
HO-1	TCCTGGCTCAGCCTCAAATG	CACGCATGGCTCAAAAACCA
Nrf-2	CCTGGAGCTTCACGTTCTCTG	TGCCACAGGATTACATCACCT

### The ability of PEDV to form resistant PEDV against luteolin on vero cells

The luteolin-resistant PEDV strains was obtained by serially passaging PEDV AH2012/12 in Vero cells, concomitant with a gradual increase in luteolin concentrations. Vero cells were initially seeded in 6-well plates and infected with PEDV at MOI = 0.05 (passage 1: P1) for 1 h, then followed by treatment with 12.5 µM luteolin for 48 h. For subsequent passaging steps, 1 mL of the virus inoculum from the previous passage was used for subsequent infections. Luteolin treatments gradually increased from 12.5 µM to 50 µM for resistance selection, with 12.5 µM increments every three passages. PEDV passaged in 0.1% DMSO (v/v) served as a mock-treated control. Reduced luteolin susceptibility (defined as a re-bounce in virus titers similar to mocked-treated controls) was determined by TCID_50_ assay on cell lysates collected at 72 h post-infection.

### Statistical analysis

Statistical analyses were performed in GraphPad Prism software (v.9.0.0) using unpaired one-tailed Student t-tests. Error bars in figures indicated standard deviation. Asterisks indicated significance levels (*P < 0.05; **P < 0.01; and ***P < 0.001) and ns = non-significant. We first filtered the differentially expressed genes (DEGs) based on adjusted *p* < 0.05 (FDR) and fold chang (FC) >  ± 1.5-fold. Differentially expressed gene (DEG) enrichment was analyzed using the Kyoto Encyclopedia of Genes (KEGG) and Gene Ontology Enrichment Analysis (GO).

### Supplementary Information

Supplementary Material 1.

## Data Availability

The original contributions to the study are included in the article material. Further inquiries can be directed to the corresponding author. And the datasets generated and/or analysed during the current study are available in the NCBI repository [accession No. PRJNA1039337].

## Abbreviations

PEDV: Porcine epidemic diarrhea virus
Mpro: Main protease
FRET: Fluorescence resonance energy transfer
SPR: Surface plasmon resonance Assay
α-CoV: Alphacoronavirus
SARS-CoV-2: Severe acute respiratory syndrome coronavirus 2
S: Spike protein
ACE2: Angiotensin-converting enzyme 2
IL: Interleukin
Vero-E6: African green monkey kidney epithelial cells
IPEC-J2: Porcine small intestinal epithelial cells
TNF-α: Tumor necrosis factor-α
MCP-1: Monocyte chemotactic protein-1
HO-1: Heme oxygenase-1
Nrf-2: Nuclear factor erythroid2-related factor 2
