# Supplementary material for: Antiviral activity of luteolin against porcine epidemic diarrhea virus in silico and in vitro
Source: BMC Vet Res. 2024 Jul 3;20:288. doi: 10.1186/s12917-024-04053-4 (PMC11221151; doi:10.1186/s12917-024-04053-4)
Supplement: Supplementary file 1 — Supplementary Material 1. [file 12917_2024_4053_MOESM1_ESM.pdf]

Original blot images:

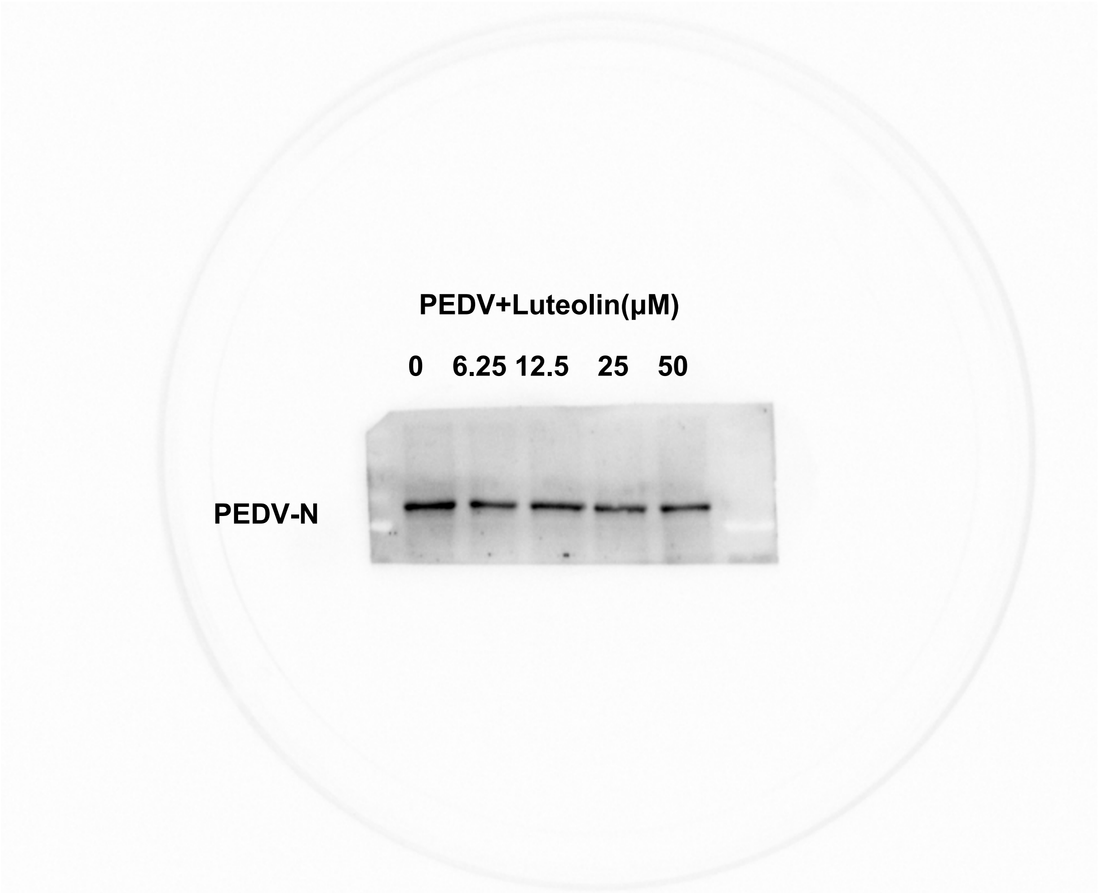

Figure 1: The expression of PEDV-N protein in Vero cells infected with PEDV and cultured with different concentrations luteolin (0, 6.25, 12.5, 25 and 50μM) by using western blot assay.

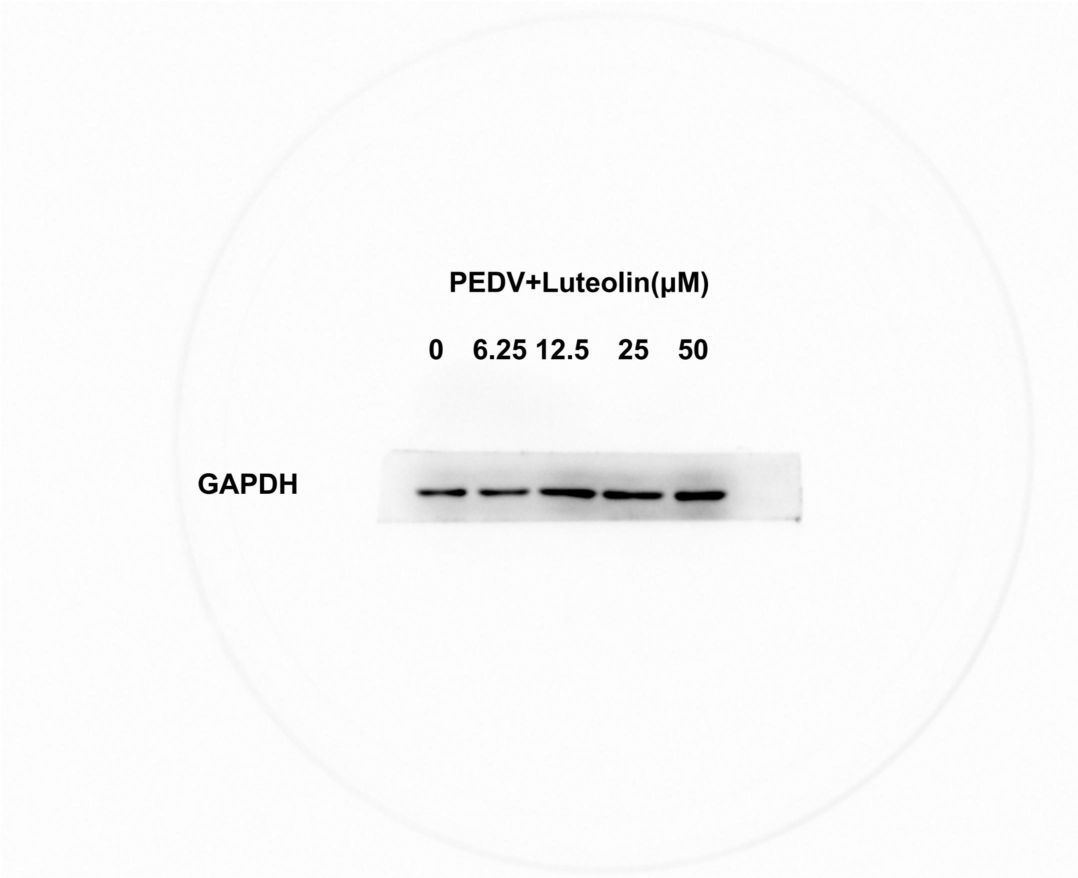

Figure 2: The expression of GAPDH protein in Vero cells infected with PEDV and cultured with different concentrations luteolin (0, 6.25, 12.5, 25 and 50μM) by using western blot assay.
